# Altered haemodynamics causes aberrations in the epicardium

**DOI:** 10.1111/joa.12977

**Published:** 2019-03-18

**Authors:** Chrysostomos Perdios, Matthew Parnall, Kar Lai Pang, Siobhan Loughna

**Affiliations:** ^1^ School of Life Sciences Medical School University of Nottingham Nottingham UK; ^2^ Present address: RDM Cardiovascular Medicine Wellcome Centre for Human Genetics Roosevelt Dr Oxford OX3 7BN UK

**Keywords:** altered haemodynamics, epicardium, extracellular matrix, heart development, outflow tract banding

## Abstract

During embryo development, the heart is the first functioning organ. Although quiescent in the adult, the epicardium is essential during development to form a normal four‐chambered heart. Epicardial‐derived cells contribute to the heart as it develops with fibroblasts and vascular smooth muscle cells. Previous studies have shown that a heartbeat is required for epicardium formation, but no study to our knowledge has shown the effects of haemodynamic changes on the epicardium. Since the aetiologies of many congenital heart defects are unknown, we suggest that an alteration in the heart's haemodynamics might provide an explanatory basis for some of them. To change the heart's haemodynamics, outflow tract (OFT) banding using a double overhang knot was performed on HH21 chick embryos, with harvesting at different developmental stages. The epicardium of the heart was phenotypically and functionally characterised using a range of techniques. Upon alteration of haemodynamics, the epicardium exhibited abnormal morphology at HH29, even though migration of epicardial cells along the surface of the heart was found to be normal between HH24 and HH28. The abnormal epicardial phenotype was exacerbated at HH35 with severe changes in the structure of the extracellular matrix (ECM). A number of genes tied to ECM production were also differentially expressed in HH29 OFT‐banded hearts, including *DDR2* and collagen XII. At HH35, the differential expression of these genes was even greater with additional downregulation of collagen I and TCF21. In this study, the epicardium was found to be severely impacted by altered haemodynamics upon OFT banding. The increased volume of the epicardium at HH29, upon OFT‐banding, and the expression changes of ECM markers were the first indicative signs of aberrations in epicardial architecture; by HH35, the phenotype had progressed. The decrease in epicardial thickness at HH35 suggests an increase in tension, with a force acting perpendicular to the surface of the epicardium. Although the developing epicardium and the blood flowing through the heart are separated by the endocardium and myocardium, the data presented here demonstrate that altering the blood flow affects the structure and molecular expression of the epicardial layer. Due to the intrinsic role the epicardium in cardiogenesis, defects in epicardial formation could have a role in the formation of a wide range of congenital heart defects.

## Introduction

The epicardium emerges from an aggregation of progenitor cells, forming the proepicardial organ (PEO), which is located inferior to the heart tube. The PEO forms around HH14 from splanchnic mesoderm as an outpouching from the septum transversum, a folding of mesodermal mesenchyme cells that give rise to the thoracic and abdominal cavities (Cano et al. 2016). These proepicardial (PE) cells migrate to the myocardium at HH17 and cover it to form the epicardium (Hiruma & Hirakow, 1989). Studies in *Xenopus* suggest that the PEO attaches to the atrioventricular canal and then proceeds to form an epicardial sheet around the heart (Tandon et al. 2013). The epicardium is of great importance, as a fraction of its cells, termed epicardium‐derived cells (EPDC), migrate into the heart and are crucial for the development of the heart and coronary vessels (Gittenberger‐de Groot et al. 1998). EPDC undergo EMT at HH19, invade the myocardium and the subendocardial region, giving rise to the subepicardial mesenchyme, or subepicardium (Lie‐Venema et al. 2005). EPDC are multipotent cardiac progenitor cells, which are important for the structural and functional integrity of the heart (Gittenberger‐de Groot et al. 1998). For example, EPDC can differentiate into vascular smooth muscle cells (SMCs) and fibroblast cells, which are important for the formation of the heart's coronary vessels and fibrous skeleton, respectively.

*WT1* is responsible for the expression of the enzyme RALDH2, which produces retinoic acid (RA; von Gise et al. 2011). RA signalling is required for the expression of *TCF21* (Braitsch et al. 2012). TCF21 is a bHLH transcription factor that is essential for EPDC differentiation into fibroblast cells while inhibiting their differentiation into SMCs, as demonstrated in a mouse null mutant (Acharya et al. 2012). Early during epicardial migration, EPDCs expressing *TCF21* are multipotent, but as development progresses, only fibroblast cells express *TCF21*. Mice with a *TCF21* knockdown lack epicardial‐derived cardiac fibroblasts, have lower collagen levels and defective EPDC migration, but normal epicardial apoptosis and proliferation (Acharya et al. 2012). In chicks, EPDC destined to become fibroblasts start invading the myocardium and the subendocardial region at HH25, with a subsequent invasion of the AV cushions at HH32 (Gittenberger‐de Groot et al. 1998).

As stated previously, one of the major cell types that develops from the migrating EPDC is fibroblast cells. As the heart develops in the embryo, the number of fibroblasts increases and so does the amount of collagen they deposit (Camelliti et al. 2005). The study of fibroblast cells is challenging due to the lack of specific markers (Goldsmith et al. 2004). *DDR2* is a receptor that binds to fibrillar collagen; although it is found in a number of cells, it is absent from cardiac endothelial cells (EC), cardiomyocytes and SMCs. The absence of *DDR2* expression in most of the heart makes it a reasonably specific fibroblast marker, with the only other cells expressing it being certain white blood cells which appear later in development (Goldsmith et al. 2004). *DDR2* null mice were found to have a smaller heart and a lower collagen density, due to slower collagen deposition, compared with the wild type (Cowling et al. 2014).

Epicardial fibroblasts are in part responsible for the production of collagen XII, a fibril‐associated collagen with interrupted triple helices (FACIT), which forms complexes with collagen I (Marro et al. 2016). Collagen XII was found in the epicardium and subepicardium of early zebrafish embryos, outlining the ventricles; as development progresses, its expression increased until it fully encases the heart and penetrates into the compact myocardium (Marro et al. 2016). Further, its expression in bone and skeletal muscle has been associated with modifying the stiffness of the tissue in response to shear stress (Chiquet et al. 2014; Marro et al. 2016).

Three main mechanical forces affect the heart: pressure, shear stress and stretch. Blood flow induces shear stress, which is a force parallel to the endocardium (Andrés‐Delgado and Mercader, 2016). Blood also creates a pressure force, which is applied perpendicular to the heart wall. Any changes in blood flow during contraction–relaxation as well as differences in blood viscosity can create a cyclic strain (Andrés‐Delgado & Mercader, 2016). Strain can cause stretching of myocardium; the factor that defines the ratio between stress and strain is the stiffness of the material. These forces can disturb normal homeostasis and result in extensive tissue remodelling (Andrés‐Delgado & Mercader, 2016).

The aim of this paper is to elucidate the effect of altered haemodynamics, by OFT‐banding, on the structure of the epicardium. The structure of the epicardium is important, as it is a multipotent progenitor of cardiac cells and changes in the ECM can affect cell migration along with other biological mechanisms (Smits et al. 2018). The epicardial morphology of OFT‐banded hearts was found to be aberrant at HH29 and HH35. The ECM alterations were mainly caused by downregulation of gene and protein expression linked to collagen and fibroblast cells. In this study, we have shown that HH29 OFT‐banded hearts have an initial epicardial phenotype with increased volume and changes in ECM expression which are exacerbated with development. By HH35, OFT‐banded hearts had increased expression of *COL12A1* and *DDR2*, which suggests a change in the ECM composition and tissue remodelling under stress.

## Methods

### Outflow tract banding

All works in this study were Schedule 1 procedures; they were ethically reviewed at the University of Nottingham and all procedures and facilities are compliant with local and institutional guidelines. *Gallus* fertilised eggs (Henry Stewart & Co., UK), of the White Leghorn variety, were placed at 38 °C in a humidified rotating incubator. At HH21, the eggs were fenestrated. The OFT‐banded embryos had their OFT constricted with a transverse, double‐overhang knot as previously published (Sedmera et al. 1999), using an Ethilon® Nylon Suture (Ethicon; W1770), which had a 3/8 circle needle attached to a 10‐0 suture. The ‘shams’ had the suture passed below the OFT but no ligature was made. Any embryos that showed haemorrhage during the banding procedure or had phenotypic malformations were excluded from the studies. The banding procedure was carried out using a Stemi SV 11 stereomicroscope (Carl Zeiss). All eggs were sealed and reincubated until HH26‐35 without rotation. Incubation times as well as the staging criteria were according to Hamburger‐Hamilton stages (Hamburger & Hamilton, 1992).

### Embryo isolation

Outflow tract‐banded and sham were isolated at required developmental stage and external analysis was performed. The OFT‐banded hearts were only processed further if the suture was still attached around the OFT with the knot intact. Any embryos with structural deformities or developmental delays were excluded from further studies. Hearts were isolated for all described studies except whole mount *in situ* hybridisation, where whole embryos were used. Whole embryos and hearts were fixed overnight in 4% paraformaldehyde (PFA) or were snap‐frozen in liquid nitrogen and kept at −80 °C.

### Processing for paraffin‐embedding and sectioning

Fixed HH29 and HH35 hearts were dehydrated in increasing concentrations of ethanol (EtOH). All hearts were sectioned at 5 μm, unless stated otherwise, anterior to posterior, on a DSC1 microtome (Leica).

### External and internal phenotypic analysis

The external phenotypic analysis of the HH29 OFT‐banded hearts (sham *n *=* *16; OFT‐banded *n *=* *16) was carried out using a stereoscope (SteREO Discovery.V8; Carl Zeiss). For internal phenotypic analysis of the HH29 OFT‐banded hearts (sham *n *=* *6; OFT‐banded *n *=* *7), sections were stained with Alcian blue (Sigma Aldrich, UK) for 15 min followed by Mayer's haemalum. Imaging was acquired with an Axioplan microscope (Zeiss). A 96‐point grid was placed over every fourth 8‐μm section throughout the HH29 embryonic hearts. Each point at which the grid hit the epicardium was counted. Once the counting was complete, the total number of counts in the epicardium was divided by the total number of counts for that heart, giving a percentage of the proportion of the epicardium contributing to the heart.

### Apoptosis and proliferation analysis

HH29 hearts (*n *=* *3 per treatment group) were serially sectioned into five groups. One of these groups was used for the apoptosis study and another group for the proliferation study. All sections were collected on superfrost plus slides (Thermo Fisher Scientific). All the stained sections were digitally captured (University of York Imaging and cytometry facility; AxioScan.Z1 slide scanner; Carl Zeiss). Visual analysis was performed using zen 2 (blue edition; Carl Zeiss) software. Positive 3,3‐diaminobenzidine (DAB)‐ and 4′,6‐diamidino‐2‐phenylindole cells (DAPI) were counted manually in the epicardium of alternating sections.

For the apoptosis study, the sections were deparaffinised in xylene and rehydrated in decreasing concentrations of alcohol. The ApopTag® Peroxidase *In Situ* Apoptosis Detection (S7100; Millipore) kit and protocol was used, with the following variations. Briefly, Proteinase K was applied to sections. Endogenous peroxidase activity was quenched using 3% hydrogen peroxidase. Equilibration buffer was applied to the sections followed by working terminal deoxynucleotidyl transferase (TdT) enzyme. Stop/wash buffer was applied on the sections, which were then incubated with anti‐digoxigenin conjugated antibody followed by 0.05% DAB with hydrogen peroxide. For counter‐staining, sections were treated with 0.5 μg mL^−1^ DAPI (Sigma Aldrich).

For the proliferation study, the sections were deparaffinised in xylene and rehydrated in decreasing concentrations of alcohol. The proliferating cell nuclear antigen (PCNA) Staining (Invitrogen) kit and protocol was used, with the following variations. Briefly, the sections were treated with H_2_O_2_, followed by blocking solution and then the anti‐PCNA primary antibody. The sections were incubated with streptavidin peroxidase followed by 0.05% DAB chromogen. They were then treated with 0.5 μg mL^−1^ DAPI (Sigma Aldrich).

### Collagen I immunohistochemistry

HH29 hearts (*n *=* *4 per treatment group) were serially sectioned into three groups. One of these groups was used for the extracellular matrix study. HH35 hearts (*n *=* *3 per treatment group) were serially sectioned into five groups. One of these groups was used for the fibroblast and extracellular matrix study.

The sections were deparaffinised and hydrated in decreasing concentrations of ethanol and permeabilised with 0.1% Triton X‐100 (Fisher). The sections were blocked with 10% normal goat serum (Invitrogen) and 1% bovine serum albumin (BSA). HH29 sections were incubated with anti‐collagen I antibodies (1 : 200; PA1‐26147; Fisher), and HH35 sections were incubated with anti‐TCF21 antibodies (1 : 200; sc377225; Santa Cruz). HH29 sections were incubated with 488 anti‐mouse antibodies (1 : 400; ab150113; Abcam), and HH35 sections were incubated with 568 anti‐rabbit antibodies (1 : 400; ab175471; Abcam). Mowiol 40‐88 (Sigma‐Aldrich) was used as mounting medium.

HH29 sections were digitally captured using an Axiovert 200M microscope (Carl Zeiss). HH35 sections were digitally captured using an AxioScan.Z1 slide scanner (Carl Zeiss; University of York Imaging facility). TCF21 analysis was performed on alternating HH35 sections using ZEN 2 (blue edition; Carl Zeiss) software, by counting the cells manually in the myocardium, epicardium and atrioventricular canal.

Twelve alternating sections from each sample, with both the epicardium and the AV canal present on each section, were chosen for further analysis. fiji (Schindelin et al. 2012) was used to quantify the void to area fraction (VAF) of collagen I by measuring the total area where collagen I was absent in the fixed area and then dividing it by the fixed area, and counting the number of cells in a region of interest (ROI). The ROI was kept at a constant area of 0.06 mm^2^ for the epicardium and 0.015 mm^2^ for the AV canal. The ROI width and height changed to accommodate the size of the epicardium.

Using the fixed area polygons, an ellipse fit was carried out, where a major and minor axis was measured in the ROI. This allowed an axes ratio measurement to be calculated using the major and minor axis (axes ratio = major axis/minor axis). As the area had a fixed size of 0.06 mm^2^ for the epicardium above the RV and 0.015 mm^2^ for the epicardium in the AV canal, any change in the major axis would be compensated by an opposite change on the minor axis. In that way, the thickness, but not the total area, of the epicardium could be measured in the two different areas.

### Western analysis

HH29 and HH35 snap‐frozen hearts (*n *=* *6 per treatment groups) were lysed using lysis buffer. The tissue was homogenised by sonication. Protein concentration was determined by Bradford protein assay (Sigma). GAPDH was used for normalisation.

The samples were run on an SDS‐PAGE gel with precision plus protein dual colour standards ladders (Bio‐Rad) transferred to a nitrocellulose membrane (Pall Corporation) and blocked using 5% BSA. Immunoblotting was performed using primary antibodies against TCF21 (1 : 750; sc377225; Santa Cruz), GAPDH (1 : 500; ab9485; Abcam), N‐cadherin (1 : 100; 6B3; DSHB) and E‐cadherin (1 : 25; 8C2; DSHB). The secondary antibodies used were horseradish peroxidase (HRP) conjugated rabbit anti‐mouse (1 : 2000; P0260; Dako) and swine anti‐rabbit (1 : 2000; P0217; Dako). Chemiluminescence was carried out using Amersham ECL Western Blotting Detection Reagents (GE Healthcare) and detected using photographic film (GE Healthcare). The photographic film was electronically scanned in TIF format. Both studies at HH29 and HH35 were repeated in triplicate. The analysis was carried out on fiji using the relative density of the pixels in each protein band, which was then normalised against GAPDH.

### RNA isolation and cDNA synthesis

Snap‐frozen hearts were homogenised in TRI Reagent® (Sigma‐Aldrich), and 1‐bromo‐3‐chloro‐propane (BCP; Sigma‐Aldrich) then added. The upper aqueous phase, after centrifugation, was mixed with 2 m pH 4 sodium acetate and isopropanol. After centrifugation, the resulting pellet was dissolved in RDD buffer with DNaseI (Qiagen). Phenol : chloroform was added and the supernatant was removed, mixed with chloroform and centrifuged again. The supernatant was removed and mixed with 3 m NaAc pH 5.2 and ethanol. After centrifugation, the resulting pellet was dissolved in DEPC‐treated H_2_O. Purity of RNA was checked using NanoDrop 2000 (Thermo Scientific).

The cDNA synthesis was performed according to the SuperScript™ II Reverse Transcriptase (RT) (Invitrogen) protocol. RT‐positive samples had SuperScript II RT added to them, and in RT‐negative samples, SuperScript II RT was replaced with dH_2_O.

### Primer design

All primers were designed using Primer‐BLAST (Ye et al. 2012), except for WT1, whose primers were sourced from the literature (Ishii et al. 2007; Supporting Information Table S1). Primers designed for *in situ* hybridisation (ISH) had a product size of 450–850 bp with various melting temperatures. Primers for qPCR had a product size of 100–230 bp and their optimal melting temperature was 62 °C.

### RT‐PCR and qPCR

The cDNA for RT‐PCR was amplified using the T100™ Thermal Cycler (Bio‐Rad). The PCR was run for 34 cycles. A 1‐kb Plus DNA Ladder (Invitrogen) was also added in one of the wells of each gel.

The standard curve was performed for each pair of primers to be used for qPCR. A duplicated, six‐point, 1 : 3 dilution series was used starting from an undiluted RT^+^, which was diluted in water control. In addition, one undiluted duplicate was used for RT^−^ and one for the water control. Relative quantification was performed with three technical repeats for each biological sample. HH29 and HH35 hearts were used (*n *=* *3 per treatment group, for both studies). A water control and an RT‐control were added for each gene of interest tested. All primers had an *R* ^2^ > 0.990 and an efficiency between 90 and 110%.

A MicroAmp® Optical 96‐Well Reaction Plate (Applied Biosystems) was used. The plate was sealed with optical adhesive film (Applied Biosystems) and the readings were taken using the 7500 Fast Real‐Time PCR System (Applied Biosystems). Analysis of the readings was done using the Pfaffl method (Pfaffl, 2001). *GAPDH* and *TBP* were used for normalisation.

### Transmission electron microscopy

HH35 hearts (*n* = 4 per group) were harvested and fixed in 3.4% glutaraldehyde diluted in 0.1 m cacodylate buffer at 4 °C. The hearts were then washed with 0.1 m cacodylate buffer. Subsequently, the hearts were post‐fixed in 1% osmium tetroxide (Agar Scientific) diluted in 0.1 m cacodylate buffer and washed in dH_2_O. The tissue was dehydrated in graded ethanol (Sigma‐Aldrich) series until it was incubated in propylene oxide (TAAB). The hearts were infiltrated with a mixture of propanol oxide and resin 3 : 1 (propanol oxide : resin) and then 1 : 1 (propanol oxide : resin). Subsequently, the hearts were infiltrated with resin and left to polymerise at 60 °C.

Sectioning took place using an EM UC6 (Leica) ultramicrotome. Samples were sectioned at a thickness of 90 nm using a diamond knife (DiATOME, TAAB) and collected on a 3.05 mm diameter copper grid (G200HH, Gilder). The sections were further stained with 50% methanolic uranyl acetate and washed briefly in 50% methanol followed by dH_2_O. Subsequently, the sections were incubated in Reynold's lead citrate solution and briefly washed in dH_2_O. Sections were visualised using a Tecnai G2 T12 BioTwin (FEI) with an accelerating voltage of 100 kV and a MegaView II (Olympus) camera system.

### Riboprobe synthesis

The GenElute™ PCR Clean‐Up (Sigma‐Aldrich) kit and protocol was used for PCR clean‐up of genes of interest (GOI) fragments, obtained by RT‐PCR. After the products had been purified, their concentration was measured with NanoDrop (Thermo Scientific). Ligation of the GOI fragments and transformation was done using the pGEM®‐T Easy Vector Systems (Promega) kit and protocol.

For the transformation, DH5α competent cells (in‐house) were used. Colonies with the right insert were midiprepped using the GenElute® Plasmid Midiprep (Sigma‐Aldrich) kit and protocol. The plasmid, in the eluate, had its concentration measured using NanoDrop (Thermo Scientific). Linearised plasmid was used as a template to synthesise digoxigenin (DIG)‐labelled RNA probes using *in vitro* transcription with SP6 or T7 RNA polymerases. The reaction was set up using Riboprobe® *in vitro* Transcription Systems (Promega) reagents and protocol. Riboprobes were purified using the ProbeQuant G‐50 micro columns (Illustra) kit and protocol.

### *In situ* hybridisation

Fixed HH24, HH26 and HH28 embryos (*n *=* *8 per treatment group) were dehydrated in increasing concentrations of methanol (MeOH). The processed embryos were stored at −20 °C for a maximum period of 2 weeks. Embryos were hydrated in decreasing concentrations of methanol. The embryos treated with Proteinase K, refixed in 4% PFA and treated with 75% post‐hybridisation buffer followed by pre‐hybridisation buffer. The ISH probes *WT1* and *TCF21* were added to the Prehybridization solution in a concentration of 530 ng μl^−1^ and left for 24 h at 65 °C.

After hybridisation, the embryos were washed with decreasing saline‐sodium citrate (SSC), followed by RNase treatment and blocking by 2% Boehringer blocking reagent (Roche) with 20% sheep serum (Sigma). Finally, embryos were incubated with anti‐digoxigenin antibody (1 : 5000; Roche) and colour development was carried out with 50% BM purple (Roche). The embryos were photographed in 100% glycerol using a Stemi SV 11 stereomicroscope (Carl Zeiss).

### Statistics

Statistics were carried using the statistical language R and its packages R commander (Fox, 2005), unless stated otherwise. GrapheR (Hervé, 2011) was used for the generation of most graphs; all the other graphs were generated in excel. The assumption of normality was tested using a Shapiro–Wilk normality test. The assumption of variance was also tested using a Levene's test for homogeneity of variance. All statistical tests were two‐tailed and had a cut‐off value of *P* < 0.05.

For qPCR, statistical analysis was done in REST (software; Pfaffl, 2002). A *t*‐test was used for the apoptosis and proliferation study, the internal phenotypic analysis and the protein expression in immunoblots. For the ROIs on the collagen 1 sections, a *t*‐test was used for the axes ratios but a Hotelling's *T* ^2^ for the number of cells and the VAF. A two‐sample Wilcoxon test was used instead of a *t*‐test when the data were not normally distributed. A two‐way anova was used to assess statistical significance of the number of TCF21^+^ cells in the different compartments of the heart. For two‐way anova, the assumption of variance was tested in the interaction of the dependent variable with the treatment and the different regions, using a Levene's test for homogeneity of variance. The Tukey test was used as the chosen *post‐hoc* test after the two‐way anova analysis.

## Results

### The epicardium of OFT‐banded hearts has an aberrant morphology at HH29

**Figure 1:**
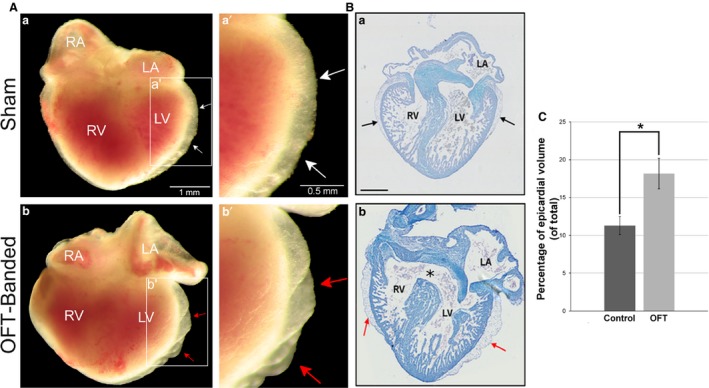
Abnormal phenotype seen in HH29 OFT‐banded hearts. (A) Controls (a,a’) displayed a normal external phenotype with an epicardium of normal size with no ruffles or blebbing (denoted by white arrow). The OFT‐banded hearts (b,b’) exhibited an enlarged epicardium with ruffles on the epicardial surface and blebbing (red arrows). Scale bars: 1 mm (a,b), 0.5 mm (a’,b’). (B) In controls (a), the interventricular septum which grows in a superior direction has fused with the cushion in the control embryos. However, this fusion failed to occur in the OFT‐banded hearts (b), which led to a formation of an opening (asterisk) and thus a communication between the ventricles (a ventricular septal defect). An aberrant epicardium can also be seen in the OFT‐banded hearts (arrows). Scale bars: 1000 μm (a,b). (C) An enlarged epicardial volume was found in OFT‐banded (OFT) hearts in comparison with shams (control). Significant differences are indicated: *P < 0.05. Error bars indicate SEM. LA, left atrium; LV, left ventricle; RA, right atrium; RV, right ventricle.

**Figure 2:**
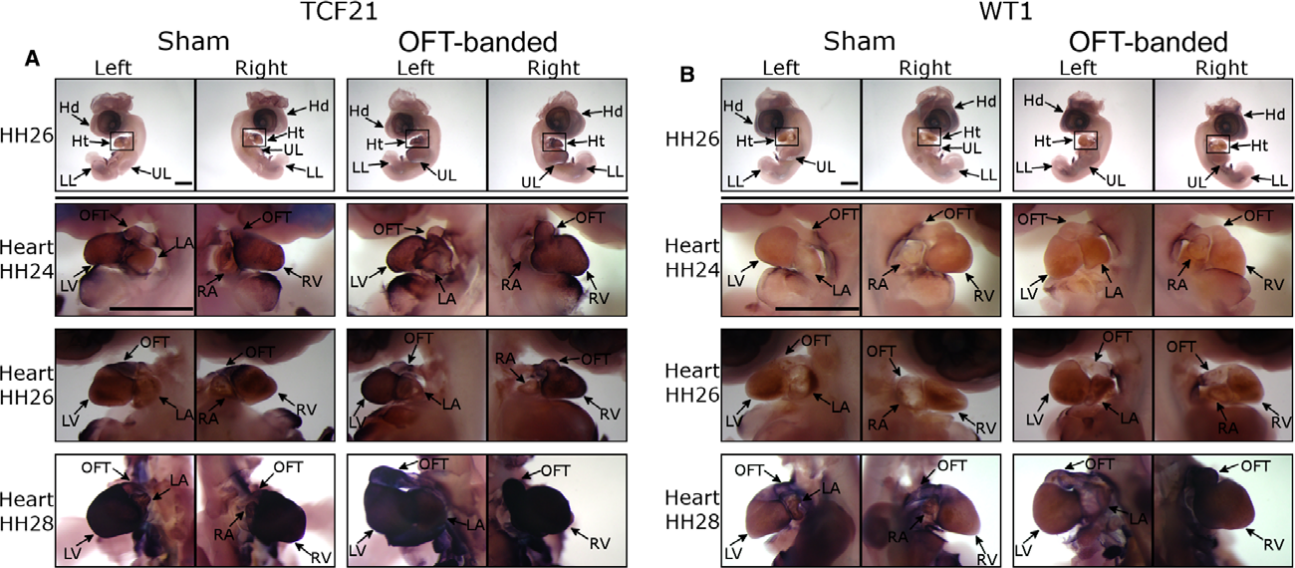
Epicardial markers were not differentially expressed in OFT‐banded hearts. (A) The expression of TCF21 around the heart region at HH24, HH26 and HH28 and a representative whole embryo at HH26. Scale bars: 2 mm. (B) The expression of WT1 around the heart region at HH24, HH26 and HH28 and a representative whole embryo at HH26. Scale bars: 2 mm. Hd, head; Ht, heart; LA, left atrium; LL, lower limb; LV, left ventricle; OFT, outflow tract; RA, right atrium; RV, right ventricle; UL, upper limb.

HH21 chicken embryos had their OFT banded by making a ligature around it, thus constricting blood flow. The chicks were reincubated up to the required stage for analysis. An impaired epicardial development was found in the OFT‐banded embryos. Increased epicardial volume could be seen in whole OFT‐banded hearts (Fig. 1Ab,Bb) in comparison with controls (Fig. 1Aa,Ba), as well as blebbing of the epicardial surface (*n *=* *10/23) at HH29 (Fig. 2Ab’,Bb) in comparison with controls (Fig. 2Aa’,Ba). No fistulae were observed between the ventricular lumen and the epicardium. To perform accurate quantification of structural features within the heart, stereology was performed (*n *=* *6 for sham, *n *=* *7 for OFT‐banded hearts). Each point at which the grid hit the epicardium was counted. For the sham and OFT‐banded hearts, a total of 4437 and 7868 points were counted, respectively. The epicardium was found to have an increased volume (total epicardial area divided by total heart area) of 60.1% in the OFT‐banded hearts (sham 11.3 ± 1.18%, OFT‐banded 18.15 ± 2.02%; *P *=* *0.02; Fig. 1C). This was in line with the increased epicardial volume phenotype found in the external morphological assessments (Fig. 2Ab,Bb).

### Normal epicardial cell migration was found upon OFT‐banding

As an aberrant epicardial phenotype was fully manifested at HH29, previous stages were selected for screening. The developmental stages chosen were HH24 (early stage of epicardial cell migration over ventricles), HH26 (epicardial formation is still active but now over the atrial region) and HH28 (epicardium formation is complete and cells migrate in the myocardium; Männer, 1999). Sixteen embryos were harvested for each developmental stage (*n *=* *8 per treatment group). The sense control for the ISH experiments can be found in Fig. S1.

The morphology of the heart was very similar between sham and OFT‐banded hearts at HH24 and HH26, with no noticeable differences in the outer morphology of the ventricles and atria (Fig. 2A,B). In contrast, in HH28 OFT‐banded hearts, the OFT was not properly aligned and wedged between the atria as in controls, with the OFT appearing to be shifted right (Fig. 2A,B). There was no apparent difference between OFT‐banded and control hearts in the expression of the epicardial markers *TCF21* (Fig. 2A) and *WT1* (Fig. 2B) in the epicardium itself. *WT1* expression (Fig. 2B) at HH24 and HH26 was lower compared with *TCF21* (Fig. 2A). At HH28, WT1 had a strong expression around the ventricles and atria (Fig. 2B). *TCF21* had the strongest gene expression; its expression increased as development progressed and it was remarkably strong at HH28 (Fig. 2A).

### Hearts at HH29 and HH35 had abnormal expression of ECM‐related genes

**Figure 3:**
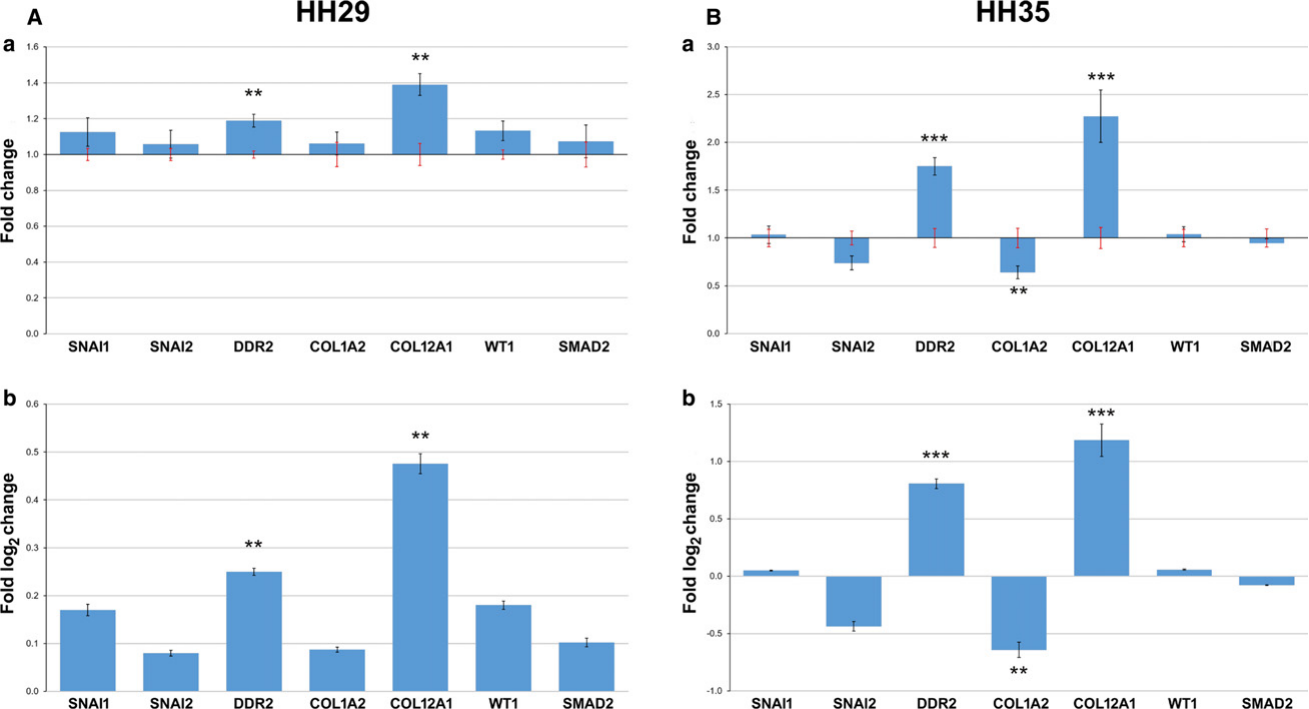
Altered expression of ECM‐related genes in OFT‐banded hearts. (A) Relative expression of genes analysed at HH29. In fold change (a), black error bars denote SEM of OFT‐banded heart gene expression, and the red error bars denote SEM of shams. The same data are also presented in log_2_ fold change (b). Significant differences: **P < 0.01; ***P < 0.001. (B) Relative expression of genes analysed at HH35. In fold change (a), black error bars denote SEM of OFT‐banded heart gene expression, whereas the red error bars denote SEM of shams. The same data are also presented in log_2_ fold change (b). Significant differences: **P < 0.01; ***P < 0.001.

A literature review was done to select genes that were found to be important in epicardial development (*SMAD2*,* WT1*), migration (*SNAI1*,* SNAI2*) and ECM structure (*DDR2*,* COL1A2*,* COL12A1*). The qPCR was performed at HH29 and HH35 hearts (*n *=* *3 per treatment group for both stages) on genes of interest. At HH29 (Fig. 3Aa,b), the genes *DDR2* and *COL12A1* were found to be significantly upregulated. *SNAI1*,* SNAI2*,* SMAD2*,* COL1A2* and *WT1* showed no significant difference (Fig. 3Aa,b). At HH35 (Fig. 3Ba,b), from the seven genes analysed by qPCR, *COL1A2* was significantly downregulated, *DDR2* and *COL12A1* were upregulated and four of the genes showed no change (*SNAI1*,* SNAI2*,* WT1* and *SMAD2*).

### Hearts at HH29 had a normal collagen I morphology and epicardial thickness

**Figure 4:**
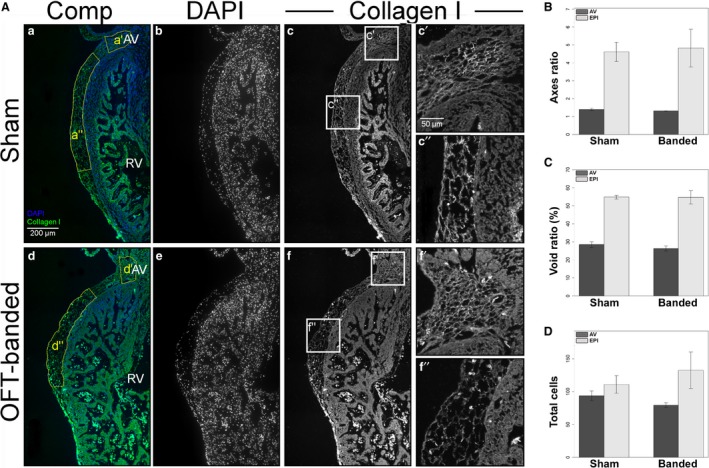
Normal collagen I expression, morphology and cell counts in HH29 hearts. (A) Hearts were stained for DAPI (b,e) and collagen I (c,f, boxes c’,c”,f’,f”). The region of interest chosen for the statistical analysis is denoted by the yellow boxes (a’,a”,d’,d”) in composite images (a,d). Collagen I morphology can be seen in detail around the atrioventricular (AV) canal (c’,f’) and above the right ventricle (c”,f”). RV, right ventricle. Scale bar: 200 μm (b–f). Scale bars: 50 μm (c’,c”, f’,f”). (B) There was no significant difference in the axes ratio of AV canal and right ventricle epicardium (EPI) region. Error bars denote SEM. (C) There was no significant difference in the void ratio of the AV canal and right ventricle epicardium region (EPI) (collagen I area/total area*100). Error bars denote SEM. (D) There was no significant difference in the cell counts of the AV canal and right ventricle epicardium region (EPI). Error bars denote SEM.

To determine the epicardial thickness and phenotypically analyse the structure of the epicardium, HH29 heart (*n *=* *4 per treatment group) sections were stained for collagen I and counterstained with DAPI to allow for quantification of collagen I area and cells (Fig. 4A). Three dependent variables were used for statistical analysis in each area, the axes ratio (Fig. 4B), the VAF (Fig. 4C) and the number of cells in the epicardium (Fig. 4D).

For the epicardial area above the AV canal (control in Fig. 4Ac,c’ in comparison with OFT‐banded in Fig. 4Af,f’), the number of cells and VAF were normally distributed. The number of cells and VAF also had equal variances. The Hotelling's *T* ^2^‐test showed no significant difference between the two treatment groups (*T* ^2^ = 2.339, *F* = 3.118, df = 3, *P* = 0.150; Fig. 4B,C). For the epicardial area above the right ventricle (control in Fig. 4Ac,c” in comparison with OFT‐banded in Fig. 4Af,f”), the number of cells and VAF were normally distributed. The number of cells and VAF also had equal variances. The Hotelling's *T* ^2^‐test showed no significant difference between the two treatment groups (*T* ^2^ = 2.191, *F* = 2.921, df = 3, *P* = 0.164; Fig. 4B,C). Apoptosis and proliferation was also normal in OFT‐banded hearts (Supporting Information Fig. S2).

In the epicardial area above the right ventricle, the axes ratio had a normal distribution and a homogeneous variance. There was no significant difference (*t* = −0.176, df = 6, *P* = 0.866; Fig. 4D) between OFT‐banded hearts (Fig. 4Af,f”) and shams (Fig. 4Ac,c’’). In the epicardial area around the AV canal, the axes ratio had a normal distribution but a significant difference in variance. There was no significant difference (*t* = −1.261, df = 4.274, *P* = 0.272; Fig. 4D) between the OFT‐banded hearts (Fig. 4Af,f’) and comparison shams (Fig. 4Ac,c’).

### OFT‐banded hearts at HH35 had an abnormal collagen I morphology and epicardial thickness

**Figure 5:**
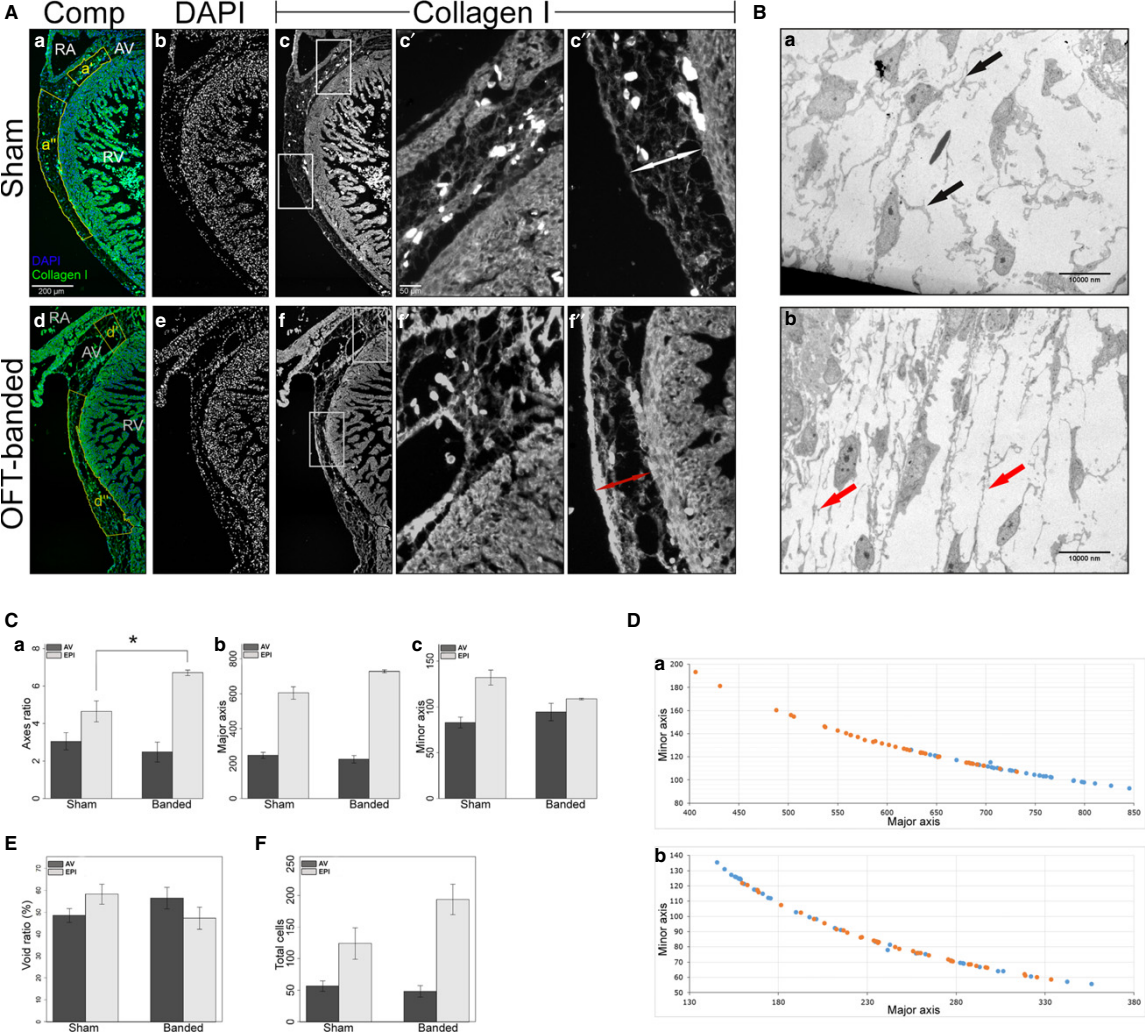
Aberrant collagen I morphology in HH35 OFT‐banded hearts. (A) Hearts were stained for DAPI (b,e) and collagen I (c,f, boxes c’,c”,f’,f”). The region of interest chosen for the statistical analysis is denoted by the yellow boxes (a’,a”,d’,d”) in composite images (a,d). Collagen I morphology can be seen in detail around the atrioventricular (AV) canal (c’,f’) and above the right ventricle (c”,f”). OFT‐banded hearts had a thinner epicardium in comparison with shams (double arrows). RA, right atrium; RV, right ventricle. Scale bars: 200 μm (a,b,c,d,e,f), 50 μm (c’,c”,f’,f”). (B) Transmission electron microscopy images of the ventricular epicardium in sham (a) and OFT‐banded (b) hearts. OFT‐banded hearts had more tense ECM fibres in comparison with shams (arrows). Scale bars: 10 000 nm (a,b). (C) There was a significant difference in the right ventricle epicardium (EPI) region, but not the AV canal, in the axes ratio (a) between sham and OFT‐banded hearts; the OFT‐banded hearts had a higher major axis (b) and a lower minor axis (c). Significant differences: *P < 0.05. Error bars denote SEM. (D) The graph shows the major to minor axis of the epicardial region above the right ventricle (a) and the AV canal (b). Orange, sham controls; blue, OFT‐banded. (E) There was no significant difference in void ratio between the AV canal and right ventricle epicardium (EPI) region (collagen I area/total area*100). Error bars denote SEM. (F) There was no significant difference in the cell counts in the AV canal and right ventricle epicardium (EPI) region. Error bars denote SEM.

To determine whether the epicardial phenotype develops in older hearts, HH35 hearts (*n *=* *3 per treatment group) were stained for collagen I and counterstained with DAPI (Fig. 5A). The epicardial morphology of HH35 hearts (*n *=* *4 per treatment group) was also further examined using transmission electron microscopy (TEM; Fig. 5B). Three dependent variables were used for statistical analysis in each area: the axes ratio (Fig. 5C,D), the VAF (Fig. 5E) and the number of cells in the epicardium (Fig. 5F).

For the epicardial area in the AV canal (control in Fig. 5Ac,c’ in comparison with OFT‐banded in Fig. 5Af,f’), the number of cells and VAF were normally distributed. The number of cells and VAF also had equal variances. The Hotelling's *T* ^2^ test showed no significant difference between the two treatment groups (*T* ^2^ = 111.751, *F* = 27.938, df = 4, *P* = 0.141; Fig. 5E,F). For the epicardial area above the right ventricle (Fig. 5Ac,c” in comparison with Fig. 5Af,f”), the number of cells and VAF were normally distributed. The number of cells and VAF also had equal variances. The Hotelling's *T* ^2^ test showed no significant difference between the two treatment groups (*T* ^2^ = 6.075, *F* = 1.519, df = 4, *P* = 0.537; Fig. 5E,F). However, the collagen I morphology of the epicardium, above the right ventricle (Fig. 5Af,f’’), was found to have a thicker fibres running along the myocardium. Upon further examination, the collagen I fibres in OFT‐banded hearts were found to be closer together (Fig. 5Af,f”), whereas the collagen fibres in shams were spaced normally (Fig. 5Ac,c”). Further examination using TEM revealed that the ECM fibres of OFT‐banded hearts were stretched anterior to posterior, parallel to the ventricular myocardium (Fig. 5Bb); in comparison, shams had relaxed ECM fibres (Fig. 5Ba).

In the epicardial area above the right ventricle, the axes ratio had a normal distribution and homogeneous variance. The OFT‐banded hearts had a significantly higher axes ratio (*t* = 3.528, df = 4, *P* = 0.024; Fig. 5Ca) in comparison with shams, denoting a thinner and wider epicardial area (Fig. 5Cb,Cc,D). In the epicardial area around the AV canal, the axes ratio had a normal distribution and homogeneous variance. There was no significant difference (*t* = −0.807, df = 4, *P* = 0.465) between the OFT‐banded hearts and shams (Fig. 5C,D).

Upon closer inspection of the major and minor axes, it can be seen that the OFT‐banded hearts’ epicardium above the right ventricle had a longer major axis (Fig. 5Cb) but a shorter minor axis (Fig. 5Cc). It was expected that the major and minor axis would have a negative linear relationship, as the area of the polygon has a fixed size. The fact that their relationship was not linear denotes changes in curvature (Fig. 5Da). The changes in the morphology of the area did not follow any kind of direction on the *z* plane (e.g. getting smaller or bigger) and were variable through the different sections of the same biological repeat.

### Hearts at HH35 had a reduced amount of TCF21 protein

To further elucidate epicardial epithelium integrity and migration as well as fibroblast development, Western analysis was performed on HH29 and HH35 hearts (*n *=* *6 per treatment group for both stages). The relative protein expression levels of N‐cadherin and E‐cadherin, markers of migration of the epicardial epithelium, along with the epicardial/fibroblast marker TCF21 were measured.

**Figure 6:**
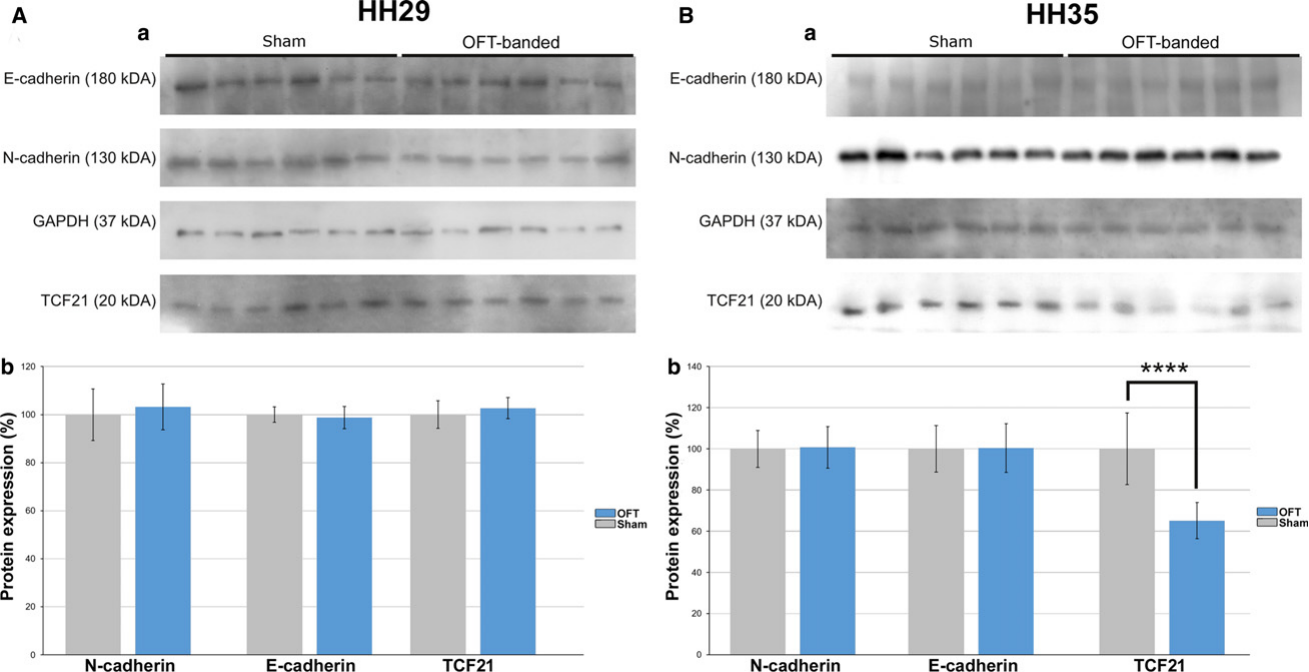
OFT‐banding reduced TCF21 protein at HH35. (A) Protein expression of E‐cadherin, N‐cadherin and TCF21 normalised to GAPDH for HH29. Intensity measurements of the bands on immunoblot (a) showed no significant difference between OFT‐banded and sham hearts (b). Error bars denote SEM. (B) Protein expression of E‐cadherin, N‐cadherin and TCF21 normalised to GAPDH for HH35. Intensity measurements of the bands on immunoblot (a) showed a significant difference between OFT‐banded and sham hearts (b) regarding TCF21. Significant difference: ****P < 0.0001. Error bars denote SEM.

For the HH29 hearts, N‐cadherin and TCF21 were normally distributed, whereas E‐cadherin was not. The assumption of variance was only tested on normally distributed dependent variables. Both N‐cadherin and TCF21 had equal variances. The cell adhesion molecules N‐cadherin (*t* = 0.511, df = 10, *P* = 0.620) and E‐cadherin (*W* = 12, *P* = 0.393) showed no significant difference (Fig. 6Aa,b). The transcription factor and epicardial marker TCF21 (*t* = 0.506, df = 10, *P* = 0.624) also showed no significant difference (Fig. 6Aa,b).

For the HH35 hearts, N‐cadherin, TCF21 and E‐cadherin were normally distributed. N‐cadherin, E‐cadherin and TCF21 also had equal variances. The cell adhesion molecules N‐cadherin (*t* = 0.161, df = 10, *P* = 0.875) and E‐cadherin (*t* = 0.043, df = 10, *P* = 0.966) showed no significant difference (Fig. 6Ba,b). The transcription factor TCF21 (*t* = −6.374, df = 10, *P* < 0.0001) showed a significant difference (Fig. 6Ba,b).

### HH35 OFT‐banded hearts have a normal number of TCF21^+^ cells

**Figure 7:**
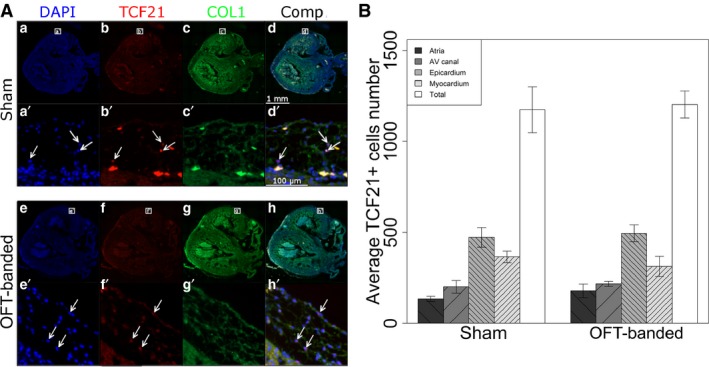
Numbers of TCF21^+^ cells are unaltered in HH35 hearts. (A) Hearts stained for DAPI (a,e), TCF21 (b,f) and collagen I (c,g). A composite can also be seen (d,h). Zoomed sections (a’,b’,d’,e’,f’,h’) show TCF21^+^ cells (arrows) as well as their collagen I surroundings (c’,g’). Scale bars: 1 mm (a,b‐h), 100 μm (a’,b’–h’). (B) No significant difference was found concerning the numbers of TCF21^+^ cells in the different heart regions between sham and OFT‐banded hearts. Error bars denote SEM. AV, atrioventricular.

The low protein expression of TCF21 together with the differential expression of collagens by qPCR led to a further investigation of the phenotype, as TCF21 is also a marker of cells destined to become fibroblasts. Six HH35 hearts (*n* = 3 per treatment group) were used for fluorescent immunohistochemistry with anti‐TCF21 and anti‐collagen I antibodies, and counter‐staining with DAPI (Fig. 7A). The average number of TCF21^+^ cells was counted in four regions: AV canal, epicardium, ventricles and atria (Fig. 7B).

The average number of TCF21^+^ cells (*W* = 0.949, *P* = 0.252) was normally distributed. The average number of TCF21^+^ cells (*F*_(df)_ = 0.538_(7,16)_, *P* = 0.794) had an equal variance. There was no significant difference between the two treatments (*F*_(1,16)_ = 0.071, *P* = 0.794) or the interaction between the treatment and the different regions (*F*_(3,16)_ = 0.571, *P* = 0.642), but there was a significant difference between the four regions (*F*_(3,16)_ = 28.239, *P* < 0.0001).

It was expected that different regions would have different amounts of TCF21^+^ cells (Fig. 7B) due to differences in region sizes and the permissiveness of epicardial migration. The epicardium had significantly more TCF21^+^ cells in comparison with the atria (*P* < 0.0001), AV canal (*P* < 0.0001) and ventricles (*P* = 0.009). The ventricles were the only other tissue to have significantly more TCF21^+^ cells and this was in comparison with the atria (*P* = 0.001). The number of TCF21^+^ cells also had a negative linear relationship with the VAF, denoting that an increased number of TCF21^+^ cells meant more collagen I in the immediate area (Supporting Information Fig. S3).

## Discussion

Within the literature, OFT‐banding is predominately performed between stages HH18 to HH21 (Sedmera et al. 1999; Midgett et al. 2014; Stovall et al. 2016). The ligature around the OFT causes a number of mechanistic effects at the OFT and within the heart, even before any major structural or genetic changes are evident. The changes seen in the OFT‐banded heart cannot be due only to the physical restriction imposed by the ligature, as the tissues above and below the band exhibit different mechanical aberrations. These mechanical responses can be observed in a matter of hours, while the embryo is still at the same HH stage, showing the adaptability of the heart (Tobita et al. 2002; Shi et al. 2013; Stovall et al. 2016). Although the motion of the ventricular wall was found to be identical between OFT‐banded and control embryos at HH18, the motion in the region of the OFT wall undergoes faster expansion and contraction movements, rather than the peristaltic‐like movements seen in controls (Stovall et al. 2016). Blood pressure, wall shear stress and pulse wave velocity (the speed of the arterial pulse in the cardiovascular system) were also found to increase in the outflow region of HH18 OFT‐banded embryos (Shi et al. 2013). In the ventricle of HH18 and HH21 OFT‐banded hearts, there was an increase in end‐diastolic blood pressure and pressure amplitude (Tobita et al. 2002; Shi et al. 2013).

In addition to the epicardium, studies have shown that a second epicardial population exists, called the arterial epicardium, which only covers the distal part of the OFT (Gittenberger‐de Groot et al. 2000; Pérez‐Pomares et al. 2003). The arterial epicardium originates from the pericardial coelom and the two epicardial populations do not make contact with each other until HH26 (Pérez‐Pomares et al. 2003). Although the arterial epicardium does not seem to make any contributions to the development of the heart, it might have a regulatory role in OFT development due to its position above the distal portion of the OFT (Pérez‐Pomares et al. 2003). Since the ligature, which resulted from the OFT‐banding procedure, is located closed to the proximal OFT, it does not affect the spreading of the two epicardial populations up to HH26. After HH26, there is the possibility that it acts as a barrier to the spreading of the arterial epicardium. As the arterial epicardium was shown to have a quiescent phenotype (Pérez‐Pomares et al. 2003), its effects on the architecture of the epicardium along the surface of the heart are considered negligible. Another point of potential interest is previous studies showing that a heartbeat is required for epicardial migration (Plavicki et al. 2014). OFT models have shown an average heart rate similar to that of controls (Menon et al. 2015), thus heartbeat was also considered to have a negligible effect in epicardial migration.

Since the epicardium is a highly heterogeneous population, no epicardial‐specific genes have been discovered (Gittenberger‐de Groot et al. 2012). Although WT1 and TCF21 have been used as the main epicardial markers, epicardial cells may not express both of them (Braitsch & Yutzey, 2013). In addition, these markers are not epicardial‐specific, as *TCF21* is also expressed in the allantois (Soulet et al. 2010) and *WT1* in the liver (Ishii et al. 2007). Although each of the main epicardial markers is by itself deemed inefficient for tracking all the epicardial cells, in this paper, *WT1* and *TCF21* were used at the same stages, using ISH, to get a clear picture of epicardial migration. At HH24, all the markers showed some weak expression over the heart but strong expression on the venous proepicardial organ, below the heart's apex, and the arterial PEO, on distal part of the OFT. Both PEOs are still present even after formation of their respective epicardial populations in the early embryo (Gittenberger‐de Groot et al. 2012).

The genes *SNAI1*,* SNAI2*,* WT1*,* COL1A2*,* COL12A1*,* DDR2* and *SMAD2* were chosen as genes of interest to further analyse quantitatively. WT1 is known to affect migration of epicardial cells through the gene *SNAI2* (Takeichi et al. 2013). *SNAI1* promotes EMT together with *SNAI2* (Medici et al. 2008). COL1A2 (collagen I) is a major structural collagen, which primarily, though not exclusively, comes from epicardium‐derived cardiac fibroblast cells (Acharya et al. 2012). COL12A1 (collagen XII) is a type of fibril associated collagen with interrupted triple helices. Collagen XII was found to be expressed in the epicardium of zebrafish (Marro et al. 2016) and binds to collagen I, changing its biomechanical properties (Koch, 1995). COL12A1 expression also changes based on tensile stress (Flück et al. 2003), with the stressed state leading to expansion. *DDR2* is a membrane receptor found in a number of cells but data suggest that in the heart it is found on cardiac fibroblasts (Morales et al. 2005). *DDR2* expression can impact collagen deposition and fibrillogenesis (Cowling et al. 2014). SMAD2 is one of the mediators of transforming growth factor β (TGFβ) signalling and it is responsible for inducing gene transcription (Nakao, 1997). TGFβ is important for the epicardium as it promotes EMT (Craig et al. 2010).

At HH29, there was upregulation of *COL12A1*, suggesting that the heart is under tensile stress, possibly due to the increased blood pressure in the ventricles. In addition, the upregulation of *DDR2* at HH29 can signal the start of altered collagen deposition in the OFT‐banded hearts and points to a signalling cascade where *DDR2* will bind to the altered collagen and further change the molecular signalling within the fibroblasts. At HH35, the further increase in expression of *COL12A1* and *DDR2*, as seen by qPCR, suggests a change in ECM composition and tissue remodelling under stress. The downregulation of *COL1A2*, at HH35, also suggests ECM remodelling.

The adhesion proteins N‐ and E‐cadherin are expressed in the cells of the epicardial epithelium (Martínez‐Estrada et al. 2010; Wu et al. 2010). An immunoblot was favoured over qPCR for the quantification of E‐ and N‐cadherin, to allow for the visualisation of any cleavage products (Ferber et al. 2008; Wheelock et al. 2008). In addition, TCF21 expression was also assessed as the only transcription factor in the epicardium with a very clearly defined role; the specification of fibroblast cells (Acharya et al. 2012). Whole uncleaved N‐ and E‐cadherin protein bands were easily visible in the immunoblot with no significant difference between HH29 sham and OFT‐banded hearts. TCF21 also showed a clear band with no significant difference between sham and OFT‐banded hearts at HH29. However, at HH35, TCF21 expression was found to be downregulated in OFT‐banded hearts. Downregulation of *TCF21* was previously associated with downregulation of collagen I expression (Acharya et al. 2012).

The number of epicardial cells also showed no significant difference between HH29 or HH35 sham and OFT‐banded hearts. These findings, together with the qPCR data showing no difference in the expression of epicardial migration markers *SNAI1* and *SNAI2* at both HH29 and HH35, suggest that epicardial migration in OFT‐banded hearts is normal. This is supported by normal expression of the epicardial markers *WT1* and *TCF21* over the surface of the heart during epicardial migration. The apoptosis and proliferation ratio was also not significantly different between sham and OFT‐banded hearts at HH29 (Supporting Information Fig. S2).

The polygons, which were used to define the affixed area at HH29 and HH35, were characterised using an ellipse fit. The ellipse fit measurements give a minor and major axis. The minor axis is always the shortest one and the major always the longest. Using the axes ratio, it is possible to measure by what approximation the shape resembles a perfect square. A perfect square is expected to have an axes ratio of 1; any deviation from 1 suggests that the shape is more elongated in one axis than the other, resulting in an elongated rectangle. Further, to quantify the total amount of collagen I in the epicardium of OFT‐banded and control hearts, the void area fraction (VAF) was determined. Although collagen I expression was not found to be significantly different in OFT‐banded hearts at HH29, the collagen I phenotype was still examined, as an aberrant expression of other ECM factors could cause a deformation of collagen I fibres. Collagen I in the epicardium of HH29 OFT‐banded hearts was found to have a normal patterning similar to shams and the epicardial thickness was found to be similar, at least in the right side of the heart. Although migration, cell number and collagen I morphology were found normal in HH29‐banded hearts, the change in the epicardial morphology could be explained by transudation of extravascular fluid from the early heart vessels coming from the sinus venosus (Poelmann et al. 1993). The extravascular fluid being pooled in the epicardium could be due to increased hydrostatic pressure in the ventricles, a well known effect of OFT‐banding (Clark et al. 1989).

HH35 OFT‐banded hearts had a higher axes ratio, in comparison with shams, meaning that the epicardium of banded hearts is more elongated along the surface of the heart and thinner. VAF analysis at HH35 showed that the amount of collagen I was unaffected in OFT‐banded hearts. However, a phenotypic difference could be seen in the arrangement of collagen I fibres in the epicardium. The collagen I fibres appeared thicker due to the epicardium being thinner, forcing the collagen fibres to group together. This ECM maturation phenotype is quite interesting, as the collagen I morphology was normal in HH29‐banded hearts. The maturation could be due to increased stiffness and stress in the hearts, as the *COL12A1* expression indicates. In addition, epicardial‐derived fibroblast cells, which appear in later stages of development (Acharya et al. 2012), could be making a greater contribution in the remodelling of the ECM at HH35. A caveat in this type of study is that the total area of the epicardium is not measured at HH35, so a higher axes ratio does not necessarily mean a smaller or larger total epicardial area. The change in the axes ratio explains the collagen I phenotype, as the same amount of collagen possibly exists in both treatments but is pressed together in the epicardium, giving it a thicker appearance. In conclusion, the epicardium at HH35 is thinner over the ventricles due to the expansion of the underlying myocardium, causing the epicardium to stretch and also to respond with an opposing force, compressing the epithelium against the myocardial surface.

The average number of TCF21^+^ cells was the same between OFT‐banded and sham hearts, which suggests that the downregulation of TCF21 did not affect the number of TCF21^+^ cells but was due to a lower expression of TCF21 in each cell. In addition, there was a positive correlation between the number of TCF21^+^ cells and the amount of collagen I. There was less void area with increased number of TCF21^+^ cells at HH35, indicating that the TCF21^+^ cells had a fibroblast fate (Fig. S3).

It is now known that a number of factors affect EPDC differentiation and migration. In addition, there is a signalling cross‐talk between the endocardium, myocardium and epicardium, where changes in one tissue can affect the development of the neighbouring layers (Lavine et al. 2005). Many of these factors have yet to be teased apart, especially the ones affecting epicardial‐derived fibroblasts (Morabito et al. 2001). Fibroblast growth factors (FGFs) are the main family of signalling molecules that are expressed by the developing myocardium and affecting epicardial EMT (Morabito et al. 2001; Vega‐Hernandez et al. 2011). FGF1, 2 and 7, in birds, were found to promote general epicardial EMT (Morabito et al. 2001) and FGF10, in mice, was found to have the ability to promote the migration of epicardial fibroblasts into the compact myocardium (Vega‐Hernandez et al. 2011). In addition, FGF9, 16 and 20 are expressed by the endocardium and epicardium, and are indispensable for normal proliferation and differentiation of epicardial cells (Lavine et al. 2005).

As the number of TCF21^+^ cells found in this study was unaltered, as well as the total number of epicardial cells, the role of FGF signalling in the epicardium, after OFT‐banding, is harder to elucidate. It is possible that an altered myocardial signalling cascade created the change in the epicardial architecture; however, we postulate that the thinning of the epicardium is due to mechanical reasons. It is well known that the ventricular area of OFT‐banded hearts is enlarged (Clark et al. 1989; Tomanek, 1999); this enlargement can result in stretching of the epicardial epithelium, adding pressure against the developing epicardium, and resulting in an equilibrium with a force perpendicular to the epicardial epithelium. The mechanistic effect does not exclude any further signalling cascades that could potentially further alter the epicardial architecture.

During myocardial infarction (MI) in the adult, the quiescent epicardium is activated and increases the expression of epicardial markers, including WT1, resulting in the migration of epicardial cells and differentiation of EPDC into fibroblasts and smooth muscle cells (Smart & Riley, 2012; van Wijk et al. 2012). In comparison with OFT‐banding, the expression of the epicardial markers *WT1* was normal at HH29 and HH35, whereas, although TCF21 expression was normal at HH29, it was decreased at HH35. Interestingly, MI models result in an increased differentiation of EPDC cells into fibroblasts; in contrast, the OFT‐banded model showed no change in the number of potential fibroblast cells (Zhou et al. 2011; van Wijk et al. 2012). A number of ECM proteins are upregulated after MI, including collagen I (Zhou et al. 2011); no upregulation of collagen I was seen in the OFT‐banded model and although the number of TCF21^+^ cells remained the same, there was an increased expression of the fibroblast marker *DDR2* and of collagen XII.

In conclusion, the abnormal epicardium morphology seen in OFT‐banded hearts is not caused by aberrant migration or cell proliferation and apoptosis but by mechanical changes in the ECM due to the change in haemodynamics. Epicardial cells respond to the mechanical changes, mainly tension, by altering the expression of a number of genes that are tied to the ECM. The upregulation of collagen XII and *DDR2* increases from HH29 to HH35, denoting a phenotype that progresses through development. By HH35, collagen I and TCF21 are downregulated and the ECM architecture shows a severe phenotype. These changes denote how a mechanical change in blood flow can cause a cascade of gene signalling in the epicardium, which is shown to affect collagen production and deposition in the epicardium.

## Conflict of interest

The authors confirm that they have no conflict of interest.

## Author contributions

M.P. optimised the protocols for ISH, RT‐PCR and qPCR and provided guidance during molecular analysis. K.L.P. developed and performed OFT‐banding, data acquisition and analysis for the internal morphological phenotyping. C.P. acquired and analysed the rest of the data and took the lead in writing the manuscript. S.L. supervised the project and provided feedback. All authors contributed to writing of the article.

## Supporting information


**Fig. S1.** Sense control for the *in situ* hybridisation study.

**Fig. S2.** Apoptosis and proliferation study.

**Fig. S3.** Relationship between void area fraction and number of TCF21^+^ cells.

**Table S1.** Primers designed for RT‐ and q‐PCR, their sequence and melting temperature (Tm).
