# Supplementary figures and images for: Altered haemodynamics causes aberrations in the epicardium
Source: J Anat. 2019 Mar 18;234(6):800–14. doi: 10.1111/joa.12977 (PMC6539700; doi:10.1111/joa.12977)

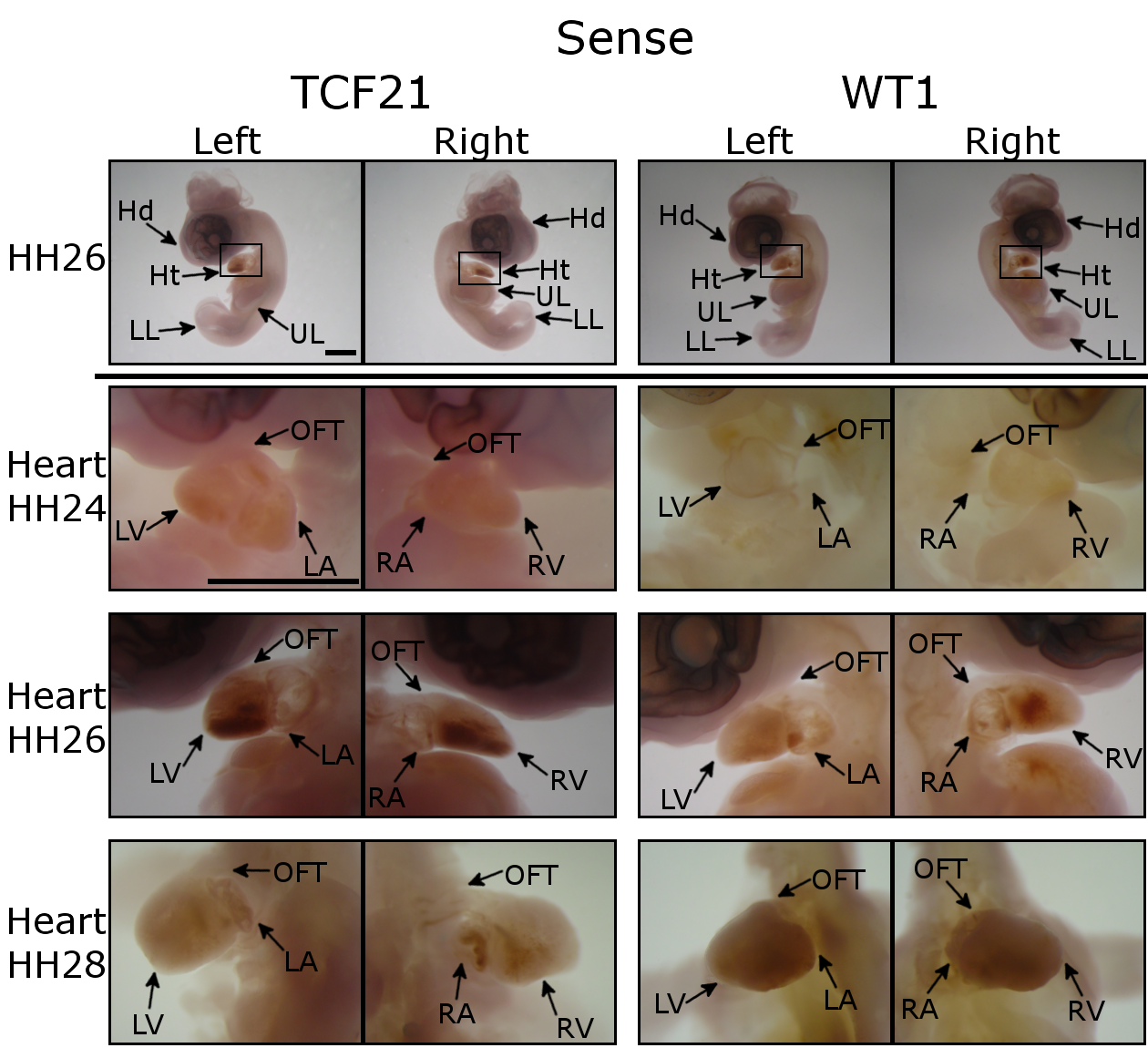

Supplement: Supplementary file 1 — Fig. S1. Sense control for the in situ hybridisation study. [file JOA-234-800-s001.tif]

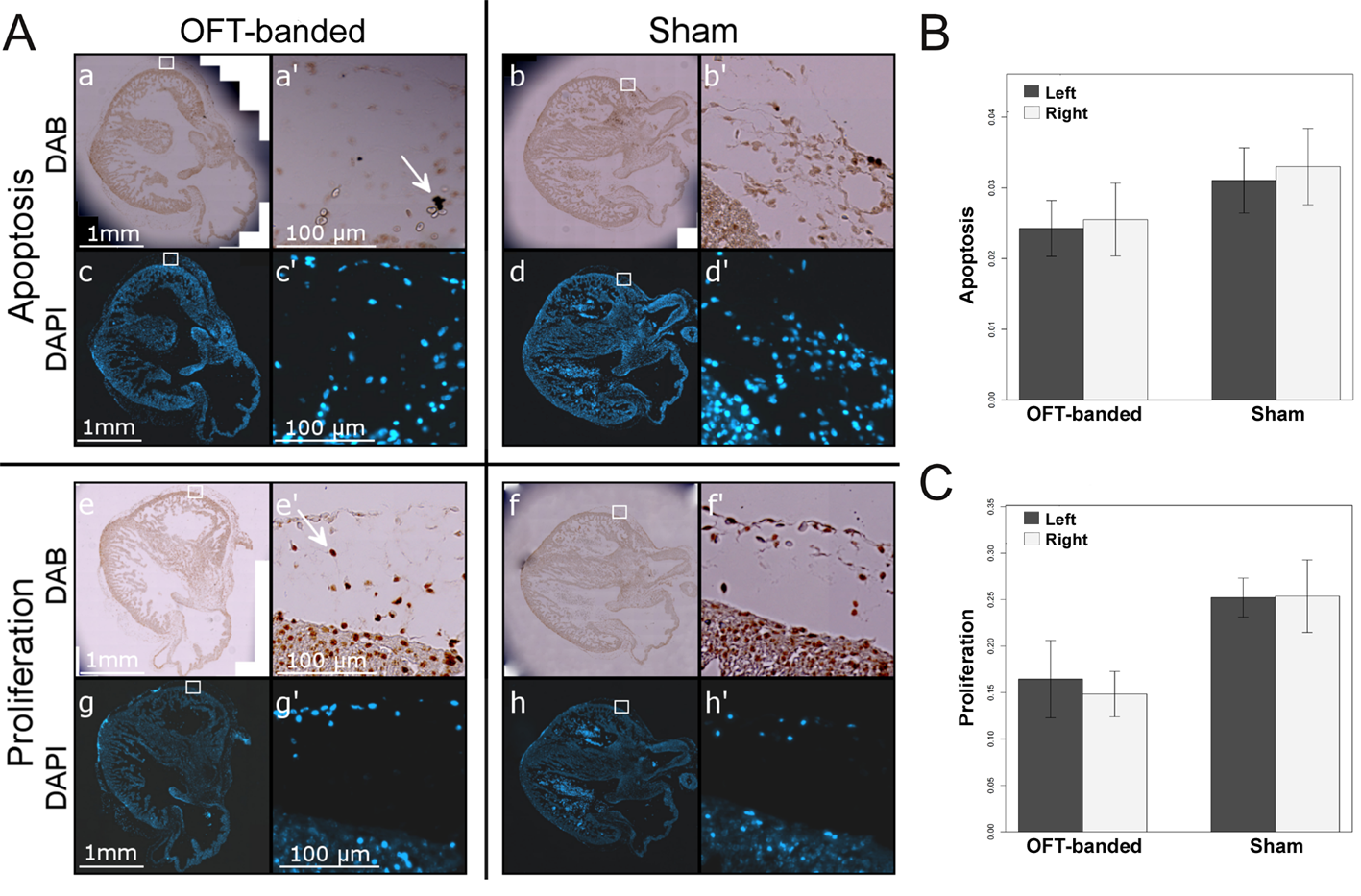

Supplement: Supplementary file 2 — Fig. S2. Apoptosis and proliferation study. [file JOA-234-800-s002.tiff]

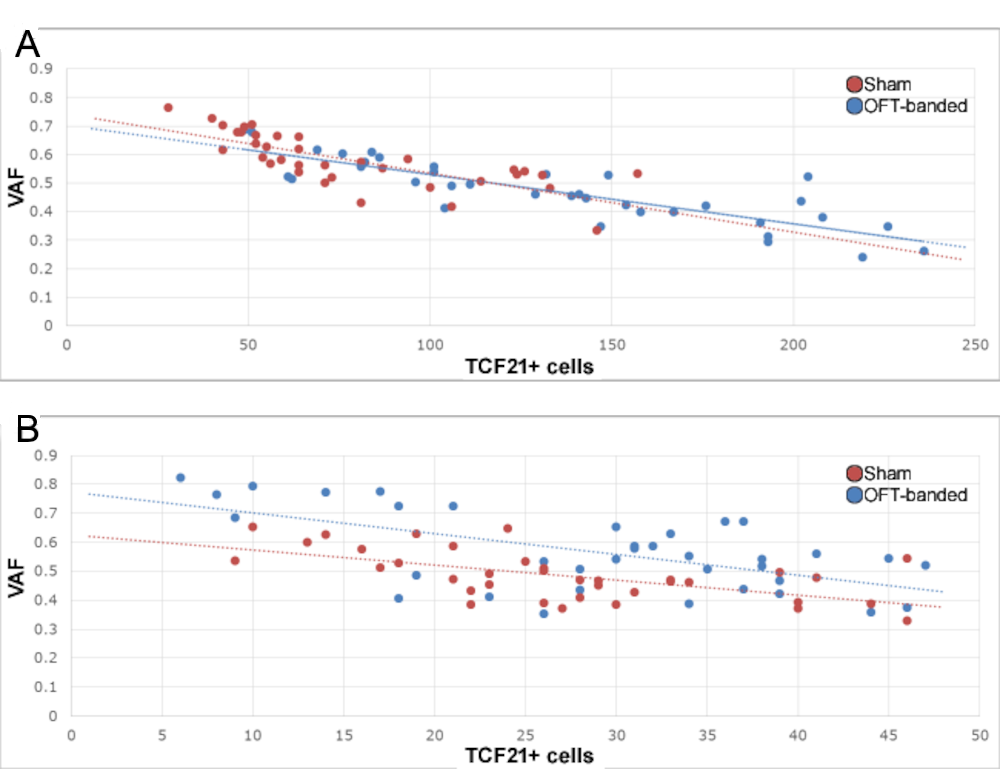

Supplement: Supplementary file 3 — Fig. S3. Relationship between void area fraction and number of TCF21+ cells. [file JOA-234-800-s003.tif]
