# Supplementary material for: Altered haemodynamics causes aberrations in the epicardium
Source: J Anat. 2019 Mar 18;234(6):800–14. doi: 10.1111/joa.12977 (PMC6539700; doi:10.1111/joa.12977)
Supplement: Supplementary file 4 — Table S1. Primers designed for RT‐ and q‐PCR, their sequence and melting temperature (Tm). [file JOA-234-800-s004.docx]

## Supplementary material

| Genes | Orientation | Sequence (5’-3') | Tm (^o^C) |
| --- | --- | --- | --- |
| *WT1** | Forward | TCTAGGGGACCAGCAGTACTC | 59 |
| (ISH) | Reverse | GATATGGTTTTTCACCAGTGTGC |  |
| *TCF21* | Forward | GTGCGATGGCCTGAAAATGG | 60 |
| (ISH) | Reverse | CGCGTGGTAGGTTTTGTTTGA |  |
| *TBP* | Forward | TGGTCAAACTCCCCAGCTCTTC | 62 |
| (qPCR) | Reverse | TTCGGGCACGAAGTGCAATG |  |
| *COL1A2* | Forward | CCTGGAGCAGATGGTAGGGT | 62 |
| (qPCR) | Reverse | GCACCTTGGTTGCCAGTGAC |  |
| *COL12A1* | Forward | TGTCATGGAAAAGGCCACCAGA | 62 |
| (qPCR) | Reverse | TGGGTTGTGCTAGGTGAAAGAGT |  |
| *GAPDH* | Forward | AGACGGTGGATGGCCCCTCT | 62 |
| (qPCR) | Reverse | ACGGCAGGTCAGGTCAACAACA |  |
| *SNAI2* | Forward | CAAAATGCCACGCTCCTTCCT | 62 |
| (qPCR) | Reverse | GGATCTCTGGCTGCGGTATGAT |  |
| *WT1* | Forward | GCCCCTTCATGTGTGCCTAC | 62 |
| (qPCR) | Reverse | GTGTCGTCTTTGGTGCCGTTT |  |
| *SMAD2* | Forward | GGGCAAGAGGAGAAGTGGTG | 62 |
| (qPCR) | Reverse | TCTGGTTTGTTCAGAGAAGCTGTAA |  |
| SNAI1 | Forward | GTACTGCGAGAAGGAGTATGTGAG | 62 |
| (qPCR) | Reverse | GCAGATTAGAACGGTCAGCAAAG |  |
| *DDR2* | Forward | CAGTGCCATCAAGTGCCAGT | 62 |
| (qPCR) | Reverse | GCGTGTGTTGCTATCGTCCA |  |
| Table 1: Primers designed for RT- and q- PCR, their sequence and melting temperature (Tm). The experiment where the primers were used are denoted; in situ hybridisation (ISH) and qPCR. * Primers taken from Ishii et al. (2007). | | | |
